# A reliable measure of frailty for a community dwelling older population

**DOI:** 10.1186/1477-7525-8-123

**Published:** 2010-10-28

**Authors:** Shahrul Kamaruzzaman, George B Ploubidis, Astrid Fletcher, Shah Ebrahim

**Affiliations:** 1 Department of Epidemiology and Population Health, London School of Hygiene and Tropical Medicine, Keppel Street, WC1E7HT, London, UK; 2 Department of Medicine, Faculty of Medicine, University of Malaya, 50603, Kuala Lumpur, Malaysia

## Abstract

**Background:**

Frailty remains an elusive concept despite many efforts to define and measure it. The difficulty in translating the clinical profile of frail elderly people into a quantifiable assessment tool is due to the complex and heterogeneous nature of their health problems. Viewing frailty as a 'latent vulnerability' in older people this study aims to derive a model based measurement of frailty and examines its internal reliability in community dwelling elderly.

**Method:**

The British Women's Heart and Health Study (BWHHS) cohort of 4286 women aged 60-79 years from 23 towns in Britain provided 35 frailty indicators expressed as binary categorical variables. These indicators were corrected for measurement error and assigned relative weights in its association with frailty. Exploratory factor analysis (EFA) reduced the data to a smaller number of factors and was subjected to confirmatory factor analysis (CFA)which restricted the model by fitting the EFA-driven structure to observed data. Cox regression analysis compared the hazard ratios for adverse outcomes of the newly developed British frailty index (FI) with a widely known FI. This process was replicated in the MRC Assessment study of older people, a larger cohort drawn from 106 general practices in Britain.

**Results:**

Seven factors explained the association between frailty indicators: physical ability, cardiac symptoms/disease, respiratory symptoms/disease, physiological measures, psychological problems, co-morbidities and visual impairment. Based on existing concepts and statistical indices of fit, frailty was best described using a General Specific Model. The British FI would serve as a better population metric than the FI as it enables people with varying degrees of frailty to be better distinguished over a wider range of scores. The British FI was a better independent predictor of all-cause mortality, hospitalization and institutionalization than the FI in both cohorts.

**Conclusions:**

Frailty is a multidimensional concept represented by a wide range of latent (not directly observed) attributes. This new measure provides more precise information than is currently recognized, of which cluster of frailty indicators are important in older people. This study could potentially improve quality of life among older people through targeted efforts in early prevention and treatment of frailty.

## Background

Identifying frail elderly people in clinical practice or in the wider population through various aspects of their health and social status is a challenge worth attempting as it would enable pre-emptive action to be taken that might avoid serious sequelae at individual and population levels. Frailty has been measured using markers such as physical ability, self reported health indicators and wellbeing, co-morbidity, physiological markers as well as psychosocial factors. Despite the efforts to quantify this experience, there is currently no standardized definition of frailty in older adults or a consensus on how it should be measured. This is evident from the numerous existing frailty measures which were driven by a common goal of reducing the burden of suffering that frailty entails - hospitalisation [1,2], falls [2-4], institutionalisation [5,6] and death [1-3,5-9]. A standardized definition could target health and social care for elderly people by enabling early detection and thereby reduce adverse outcomes and costs of care. This may also lead to more effective strategies to prevent or delay the onset of frailty as well as interventions that target the 'pre-frail elderly' or those at high risk of becoming frail. These efforts would be aimed at improving the quality of life of older people.

The current situation has evolved where "frailty" is used without a standardized definition, measured in a variety of ways and for a range of purposes [10]. The lack of consensus is reflected in three types of measures that exist in literature - rules based, clinical judgement and indexes [11]. The first determined that frailty was made up of a set number of criteria. Fried's rules-based frailty criteria as validated by other studies [1,3,7], give primacy to physical measures of frailty. Other measures assume a multi-dimensional form [12-14] or, at the other extreme, a single component physical/physiological measure such as grip strength [15], walking speed [16], functional reach [17] and blood markers [18,19]. Frailty measures relying on clinical judgement to interpret results of history taking and clinical examination are unlikely to be repeatable and will vary from clinician to clinician making them of little value for research or audit purposes[6]. The frailty index approach is based on a proportion of deficits accumulated in an individual in relation to age [20,21]. The problem with this measure is the use of 'unweighted' variables that assume that deficits such as 'cancer' and 'arthritis' are of equal importance to one another in indexing frailty. Also, in large indexes (40 or more variables) a smaller subset of items, selected at random, were similarly associated with the risk of adverse outcomes as the whole set of items [21]. The more variables considered, the greater the problems of measurement error and missing data. Despite its reproducibility, [22,23] and high correlation with mortality [5,21], the index measure is time consuming and not widely used clinically. Additionally, all three types of measures may not be measuring frailty alone but also comprise other entities that overlap with frailty such as morbidity or disability. Although these frailty measures provide useful information on frailty markers from clinical and physiological characteristics that show strong correlation with the risk of adverse outcomes, a standardized measure of frailty would be better placed to provide adequate evidence to inform policy and clinical practice.

To date, no model of frailty based on defining and quantifying frailty on a purely data driven approach has been produced. Thus we propose a frailty model developed from factor analysis (FA), a robust analytical technique which uses latent variables as a means of data reduction to represent a wide range of attributes/variability among observed variables on a smaller number of dimensions or factors[24]. These latent factors are not directly observed but rather inferred (through a statistical model) from directly observed or measured variables[25]. This mirrors the concept of frailty as a latent vulnerability in older adults, subtle, often asymptomatic and only evident over time when excess vulnerability to stressors reduces the older person's ability to maintain or regain their homeostasis[26]. Our model's advantage over previous frailty measures is that it corrects for measurement error and assigns relative weights in the association of each indicator with frailty. By controlling for measurement error, this method tested the assumption of whether the frailty measure is uni-dimensional or not. Potential sources of the amount of error, both random and systematic inherent in any measurement can range from the mistaken or biased response of patients on self rated health questionnaires to the error of measurement when taking their weight, height or blood pressure

In this paper we develop a model- based measure of frailty and examine its reliability for use in a community dwelling elderly population. We also compared the predictive ability of this new frailty measure with a widely known frailty index[27] in relation to adverse outcomes such as all cause mortality, time to hospitalization and institutionalization.

## Method

### Data and study population

The British Women's Heart and Health Study (BWHHS) cohort of women provide the dataset for the construct of frailty. Its methodology has been fully described elsewhere[28]. Briefly, between 1999 to 2001, a cohort of 4286 women aged 60-79 years was recruited from general practice lists in 23 nationally representative UK towns. Participants attended an interview where they were asked about diagnosed diseases and underwent a medical examination that recorded blood pressure, waist and hip circumference, height and weight. The women completed a questionnaire collecting behavioural and lifestyle data, including smoking habit, alcohol consumption and indicators of socio-economic position.

Thirty five (35) indicators represented a multidimensional view of frailty incorporating its physical, physiological, psychological and social aspects. These frailty indicators included those in existing literature [11,13,20,26,27,29,30] that were also available in the dataset. These included variables derived from self-reports of health status, diseases, symptoms and signs, social as well as lifestyle indicators (see Additional file 1: Supplementary Table S1). Blood investigations (see Additional file 1: Supplementary Table S2) were deliberately excluded to create a measure that was non- invasive and practical to identify elderly people at risk in a primary care setting. These were extracted from the BWHHS database and recoded into binary categorical variables.

This model derived from the BWHHS data was replicated using data from the "usual care" arm of a large randomised trial of health care in general practice for people aged 75 and over. General practices from the MRC General Practice Research Framework were recruited to the trial[31]. The sampling of practices was stratified by tertiles of the standardized mortality ratio (mortality experience of a local area relative to the national mortality) and the Jarman score [32] (a measure of area deprivation) to ensure a representative sample of the mortality experience and deprivation levels of general practices in the United Kingdom. Practices were randomly assigned to two groups receiving targeted or universal screening. All participants received a brief multidimensional assessment followed, in the universal arm by a nurse led in-depth assessment while in the targeted arm the in-depth assessment was offered only to participants with pre-determined problems at the brief assessment. The in depth assessment included a wide range of health related, social and psychological factors while in the targeted arm only elected patients had a full assessment. The baseline assessments were performed between 1995 and 1999. In these analyses we used data only from participants in the universal arm (53 practices) as they were considered a representative sample of community dwelling older people receiving "usual" care. People living in nursing homes were not eligible for the trial. This study has approval from the 23 Local Research Ethics Committees covering our BWHHS study population. All women gave signed informed consent at baseline. Local Research Ethics Committee approvals were similarly obtained for all the practices participating in the MRC trial.

In both cohorts, a complete case was defined as those respondents with complete data on all 35 frailty indicators. There were 4286 women respondents from the BWHHS database of which 1568 had complete data. People in the MRC replication dataset comprised 9032 women (6709 complete data) and 5622 men (4486 complete data).

Since their time of entry into the study until the censored date of 10^th ^August 2008, there were 633 deaths among the BWHHS study cohort giving a median follow up period of 8.2 years (range 4 months to 9.3 years). In the MRC assessment study, since their entry into the study until the 4th of October 2007, 7469 out of 11195 respondents of the MRC Assessment study have died (66.7%). Of the 6709 women, 4197 had died (62.6%). Of the 4486 men, 3272 had died (72.9%). In the mortality analysis, all MRC respondents were followed up for a median time of 7.9 years (range 22 days to 12.6 years. When 'time to first hospital admission' was used as the outcome measure, the MRC respondents were followed up for a median time of 2 years (range 22 days to 2 years). This shorter follow up period for hospitalization data was because these data were not collected for the full duration of follow up. For similar reasons, in the analysis using admission into an institution as the outcome measure, all MRC respondents were followed up for a median time of 3.9 years (range 1.6 to 5.7 years).

### Statistical analysis: Factor analysis with Exploratory Factor Analysis (EFA) and Confirmatory Factor Analysis (CFA)

**Figure 1:**
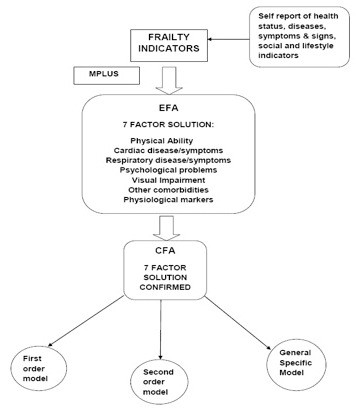
**Overview of steps in factor analysis using the BWHHS frailty indicators**.

In order to better define frailty, factor analysis (FA) appropriate for binary data was conducted using the Mplus software (version 4.2). FA is a statistical technique used to analyze correlations among a wide range of observed variables to explain these variables, largely or entirely, in terms of their common underlying (latent) dimensions called *factors*, in this case, frailty[24]. EFA was used to explore the underlying factor structure of the frailty indicators and develop the construct/hypothesis of frailty. The resulting EFA model was subjected to CFA to further test this latent structure. We proceeded by testing the higher order dimensionality of the EFA driven 1^st ^order solution by estimating a 2^nd ^order and a general specific model. In EFA as well as the three CFA models (1^st ^order, 2nd order and General Specific Models), Mplus initially estimated the factor loadings and item thresholds. Standardised factor loadings can be thought of as the correlation of the original/manifest variable (frailty indicator) with a latent factor and are useful in determining the importance of the original variable to the factor. Item threshold refers to the level of the latent factor (i.e. frailty) that needs to be attained for a response shift in the observed variables. Although the response scale for each frailty indicator is binary (1 "present" or 0 "absent"), the underlying factor model assumes that each indicator varies on an underlying continuous scale and each person can be located on that continuum[33]. Persons located above a certain threshold on that continuum will endorse that the frailty indicator was present. Each of these possible measurement models were analyzed to see which best fit the data as well as the concept of frailty. Figure 1 gives an overview of the steps taken in factor analysis.

Factor analysis was carried out on respondents with complete data on all 35 frailty indicators, which resulted in a study population of 1568 complete cases, as well as the total study population of 4286 women which included those with partial data (i.e. those with at least one frailty indicator missing). In addressing the problem of missing data in the frailty indicators used in the analysis, the model was estimated with the WLSMV (Weighted Least Squares, Mean and Variance adjusted) which applies pair-wise missing data analysis using all individuals with observations for all possible pairs of variables in the data. Individuals with partial data are therefore retained in the analyses and their information was used for all further analyses. In our case, the pairs are frailty items. A sensitivity analysis using an ***unpaired t-test ***was carried out to compare the mean difference between the complete case frailty score of 1568 women and the frailty scores of the total population of 4286 women with missing frailty indicators included. At a 5% level, the difference in means was not significant with a p value of 0.54, showing no difference in mean scores derived from both groups. Hence further analysis was carried out using the total BWHHS study population of 4286 women

In both datasets, complete cases were compared to cases with missing data, by looking at goodness of fit indices and at factor loadings in each dataset. In the model of choice, the derived factor score for frailty (i.e. scores of a subject on the frailty factor) was examined to explore the distribution of frailty by age and/sex in each study population.

### Goodness of fit test

The Scree plot approach, the Kaiser-Guttman rule (for EFA only) and indices of fit such as the Comparative Fit Index (CFI), the Tucker Lewis Index (TLI) and the Root Mean Square Error of Approximation (RMSEA) (for both EFA and CFA) were used as a means of evaluating results of the FA. Both the *Scree plot *and *Kaiser-Guttman rule *was used to decide on the number of factors/dimensions to be retained for further analysis[34]. The *Scree plot *is a graph of each eigen value which represents the total variance of each factor, (Y-axis) against the factor with which it is associated (X-axis). The *Kaiser Guttman rule *retains only factors with eigen value larger than 1[34]. The *CFI *refers to the discrepancy function adjusted for sample size. *TLI *was used to assess the incremental fit of a model compared to a null model. Both range from 0 to 1 with a larger value indicating better model fit. Acceptable model fit is indicated by a *CFI *and *TLI *value of 0.95 or greater. *RMSEA *is related to residual in the model. *RMSEA *values range from 0 to 1 where an acceptable model fit is indicated by an *RMSEA *value of 0.06 or less. A chi-squared goodness of fit test and these indices of fit were used to assess model fit as suggested by guidelines proposed by Hu and Bentler [35]. These goodness of fit indices were emphasized since the chi-squared test was deemed highly sensitive to sample size, leading to rejection of well-fitting models.

### Comparison of the new frailty measure with a widely known frailty index

We compared the predictive ability of our new measure, the British frailty index (BFI), with the Canadian Study of Health and Aging (**CSHA) frailty index**[27]. Apart from being closely related to a more multi dimensional concept of frailty, the CSHA index is one of the most widely published frailty measures, having been evaluated in many study populations [22,36-38]. The CSHA frailty index was calculated as the proportion (from a given set) of deficits present in a given individual, and indicating the likelihood that frailty was present. The ranges of deficits were counted from variables collected from self-reports or clinically designated symptoms, signs, disease and disabilities that were readily available in survey or clinical data. The variables for each FI were recoded as binary with value '1' when the deficit was present and '0' when absent. For example, if a total of 20 deficits were considered, and the individual had 3, then the frailty index value is 3/20 = 0.15.

FI = X/Y = Sum of deficits/total number of variables

Using the equation above, the CSHA frailty index was developed using unweighted variables from the BWHHS and MRC assessment study datasets. The difference between the variables included in the CSHA FI and those used when developing the BFI are given in Additional file 1. This identifies the more important and higher weighted variables in the BFI that were derived from factor analysis and allows us to differentiate it from the unweighted CSHA FI.

### Cox regression analysis

Cox proportional hazards regression analysis was used to compare the difference between hazard ratios for adverse outcomes when using the British FI and the CSHA frailty index. Hazard ratios for all cause mortality were compared in both the BWHHS and MRC assessment study datasets and risk of first hospital admission and institutionalization was assessed using data that was only available in the MRC assessment study.

As there was no violation of the proportional hazards assumption in the BWHHS dataset, the hazard ratio for all cause mortality was calculated for the whole follow up period ranging from 4 months up to 9.3 years. However, the assumption of non-proportional hazards was violated in the MRC assessment study. To fulfill the assumption of proportional hazards, the analysis time was split or divided into three shorter time periods: 0 to 2.5 years, 2.5 to 5.5 years and 5.5 to 12.6 years (end of follow up time).

In both datasets, the covariates introduced into the Cox regression model were age, sex (MRC study only), marital status, housing tenure, living alone or otherwise, social contact (good or poor), smoking, alcohol intake and socioeconomic position (SEP) scores (BWHHS only). Crude, partially adjusted (age and/or sex) and fully adjusted models were fitted for these outcomes. To address the problem of missing data in the BWHHS covariates that were adjusted for in the Cox regression model, a multiple imputation procedure provided unbiased estimates of the parameters and their standard errors in the model. This was not necessary for the MRC assessment covariates adjusted for, as they had less than 2% missing data.

## Results

### Exploratory factor analysis (EFA)

Seven factors were needed to adequately explain the association between the frailty indicators and were labelled as: *physical ability, cardiac disease or symptoms, respiratory disease or symptoms, physiological measures, psychological problems, co morbidity *and *visual impairment*.

Each of these identified latent factors was derived from subsets of indicators that correlated strongly with each other and weakly with other indicators in the dataset. They provided meaningful theoretical 'explanations' or 'interpretations' linking them to the overall construct of frailty. *'Physical ability' *comprised of highly correlated indicators such as level of activity, ability to do household chores, go up and downstairs, walk out and about wash, dress or groom oneself. '*Cardiac and respiratory disease or symptoms' *included self report or doctor diagnosis of myocardial infarction, angina, asthma, chronic obstructive airways disease or emphysema and their associated symptoms of chest pain or discomfort, pain on uphill or level walking, shortness of breath, increase cough or frequent wheeze. The '*physiological measures' *included body mass index (BMI), waist hip ratio (WHR), pulse rate, blood pressure as well as evidence of orthostatic hypotension. Markers such as subjective feelings of anxiety or depression, self reports and diagnosis of memory problems and depression were meaningfully explained by '*psychological problems'*. Other indicators such as stroke, diabetes, hypertension, peptic ulcers, thyroid disease and cancer were also explained by '*comorbidity'*. Lastly, *'visual impairment' *explained the correlations between indicators of diagnosed cataract or glaucoma as well as a self-report of visual problems.

### Confirmatory Factor Analysis (CFA)

**Figure 2:**
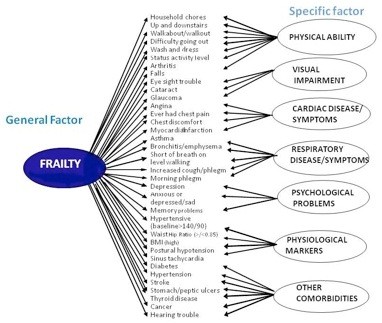
**The General Specific Model**.

We empirically compared three latent structures based on the EFA seven factor model: 1st order, 2nd order and General specific models. Model fit statistics for each of the models tested in both BWHHS and MRC datasets are shown in Table 1. These results support the contention that the frailty model of choice for both BWHHS women and the MRC Assessment study (both men and women) was the ***General Specific model ***(see Figure 2). **General **refers to frailty, the general factor that is loaded (explained by) all the indicators. **Specific **refer to the 7 latent factors that account for the association between the frailty indicators and the specific dimensions/factors. The fit of the General Specific frailty model was better than each of the other two models (see Additional file 1: Supplementary figure F1: First order model and Supplementary figure F2: Second order model) in both datasets. This was true for participants with complete data as well as those with missing data, with very little difference between them.

**Table 1:** Results from confirmatory factor analysis for the BWHHS and MRC Assessment Study (Complete cases and Missing)

**CFA 1**^**st **^**ORDER MODEL**						
**Indices of Model Fit**	**BWHHS Complete Cases (FEMALE)**	**BWHHS Missing** **(FEMALE)**	**MRC Complete Cases (FEMALE)**	**MRC Missing (FEMALE)**	**MRC Complete Cases (MALE)**	**MRC Missing (MALE)**

**X^2^**	6404.29	22275	42380	76468	23473	39003

**df**	195	251	292	290	266	264

**p**	0.000	0.000	0.000	0.000	0.000	0.000

**CFI**	0.938	0.932	0.962	0.968	0.941	0.962

**TLI**	0.949	0.950	0.970	0.976	0.955	0.972

**RMSEA**	0.032	0.032	0.025	0.027	0.029	0.027

**CFA 2**^**nd **^**ORDER MODEL**						

**Indices of Model Fit**	**BWHHS Complete Cases (FEMALE)**	**BWHHS Missing** **(FEMALE)**	**MRC Complete Cases (FEMALE)**	**MRC Missing (FEMALE)**	**MRC Complete Cases (MALE)**	**MRC Missing (MALE)**

**X^2^**	6404	22275	42380	76468	1820	39003

**df**	195	251	292	290	355	264

**p**	0.000	0.000	0.000	0.000	0.000	0.000

**CFI**	0.931	0.925	0.954	0.960	0.937	0.957

**TLI**	0.944	0.946	0.965	0.970	0.953	0.969

**RMSEA**	0.034	0.033	0.027	0.029	0.030	0.028

**GENERAL SPECIFIC MODEL**						

**Indices of Model Fit**	**BWHHS Complete Cases (FEMALE)**	**BWHHS Missing** **(FEMALE)**	**MRC Complete Cases (FEMALE)**	**MRC Missing (FEMALE)**	**MRC Complete Cases (MALE)**	**MRC Missing (MALE)**

**X^2^**	6404	22275	42380	76468	23473	39003

**df**	195	251	292	290	266	264

**p**	0.000	0.000	0.000	0.000	0.000	0.000

**CFI**	**0.957**	**0.948**	**0.967**	**0.969**	**0.954**	**0.970**

**TLI**	**0.964**	**0.962**	**0.974**	**0.976**	**0.964**	**0.978**

**RMSEA**	**0.027**	**0.028**	**0.024**	**0.026**	**0.026**	**0.024**

Cut off criteria for good fit- CFI&TLI > 0.95, RMSEA < 0.06- Hu and Bentler 1990.

In the BWHHS complete data, standardized factor loadings of the frailty indicators by the overall Frailty factor (i.e. correlations of the observed frailty indicators with Frailty) revealed highest loadings (0.60-0.77) on indicators such as being 'short of breath on level walking', the inability to do 'household chores', 'walking up and down stairs', 'walking about', 'wash and dress',' having a low 'status activity level' as well as 'difficulty going out'. This is followed by midrange loadings (0.3-0.55) of having symptoms of 'angina', 'chest discomfort' or 'ever having chest pain', 'arthritis',' feeling 'anxious or depressed', 'memory problems', having a 'high body mass index (BMI)' or 'waist hip ratio', 'eyesight trouble', 'hearing trouble' as well as having specific diseases (see Table 2). These 'weighted' loadings form the basis of an idea for which indicator would be useful to include in a frailty measure. When replicated in the MRC complete dataset of women, these factor loadings were similar to the BWHHS dataset. Factor loadings for 'hypertension' and 'waist hip ratio' by overall frailty were lower in men compared to women in the MRC dataset.

**Table 2:** Standardized Factor loadings of the general/overall Frailty factor derived from the General Specific model in both the BWHHS and the MRC Assessment study

Variable factor Loadings:	BWHHS complete cases	BWHHS Missing	MRC female Complete cases	MRC Female missing	MRC Male Complete cases	MRC Male missing
Household chores	0.736	0.759	0.632	0.722	0.718	0.765

Up and downstairs	0.725	0.748	0.739	0.800	0.791	0.808

Walkabout/walkout	0.685	0.673	0.745	0.821	0.865	0.878

Difficulty going out	0.601	0.635				

Wash and/or dress	0.612	0.594	0.592/0.521	0.683/0.620	0.657/0.604	0.712/0.685

Status activity level	0.616	0.585	0.655	0.731	0.746	0.785

Arthritis	0.421	0.434	0.324	0.322	0.176	0.206

Falls	0.261	0.390	0.342	0.389	0.387	0.444

Eye sight trouble	0.410	0.385	0.485	0.486	0.438	0.467

Cataract	0.325	0.305	0.229	0.201	0.180	0.186

Glaucoma	0.195	0.158	0.054	0.063	0.065	0.031

Angina	0.550	0.587				

Ever had chest pain	0.401	0.413	0.287	0.254	0.274	0.250

Chest discomfort	0.405	0.482	0.331	0.279	0.341	0.297

Myocardial Infarction	0.344	0.433	0.303	0.281	0.310	0.273

Asthma	0.263	0.347	0.196	0.154	0.224	0.201

Bronchitis/emphysema	0.260	0.320	0.336	0.284	0.369	0.311

Short of breath on level walking	0.770	0.815	0.676	0.624	0.699	0.683

Increased cough/phlegm	0.247	0.303	0.193	0.150	0.220	0.220

Morning phlegm	0.305	0.394	0.267	0.231	0.281	0.278

Depression	0.300	0.390	0.172	0.150	0.214	0.195

Anxious or depressed/sad	0.418	0.462	0.426	0.405	0.367	0.404

Memory problems	0.365	0.399	0.349	0.354	0.396	0.447

Hypertensive (baseline > 140/90)	0.036	-0.009	-0.054	-0.076	-0.110	-0.116

Waist Hip Ratio (>/< 0.85)	0.362	0.262	0.228	0.278	0.034	0.040

BMI (high)	0.412	0.346	0.342	0.420	0.232	0.348

Postural hypotension	0.114	0.048	-0.020	-0.009	0.046	0.060

Sinus tachycardia	0.111	0.058	-0.030	-0.028	0.120	0.102

Diabetes	0.305	0.244	0.196	0.196	0.178	0.205

Hypertension	0.340	0.304	0.110	0.060	0.090	0.064

Stroke	0.412	0.403	0.372	0.411	0.402	0.432

Stomach/peptic ulcers	0.241	0.340	0.258	0.196	0.120	0.103

Thyroid disease	0.191	0.250	0.143	0.104	-0.090	0.095

Cancer	0.150	0.072	0.033	0.014	0.042	0.018

Hearing trouble	0.310	0.344	0.357	0.337	0.265	0.290

In the general specific model, the standardized factor loadings of frailty indicators on the seven specific latent factors (correlation of individual frailty indicators with each specific factor), are shown in Table 3. These loadings show how differently the frailty indicators correlate with frailty, compared to their specific factors. The differences in the values reflect the degree of correlation of the variable with either factor, for example; the variable 'angina' has a factor loading of 0.550 on the general (frailty) factor and a loading of 0.619 on its specific factor (Cardiovascular symptoms/disease) with both factors independent of each other. Hence although 'angina' loads highly under its specific factor, its correlation with frailty in relation to all other variables is lower. The model produced individual frailty scores for all subjects in each dataset.

**Table 3:** Standardized factor loadings of specific factors derived from the General Specific model

Specific Factors	BWHHS complete cases	BWHHS Missing	MRC female Complete cases	MRC Female missing	MRC Male Complete cases	MRC Male missing
**Physical Ability**						
						
Household chores	0.533	0.524	0.624	0.561	0.500	0.477
Up and downstairs	0.557	0.532	0.483	0.414	0.399	0.378
Walkabout/walkout	0.622	0.627	0.562	0.459	0.366	0.343
Difficulty going out	0.622	0.581				
Wash and/or dress	0.635	0.627	0.641/0.632	0.577/0.602	0.657/0.604	0.605/0.540
Status activity level	0.217	0.263	0.470	0.411	0.746	0.274
Arthritis	0.372	0.356	0.106	0.043	0.176	0.115
Falls	0.104	0.097	0.179	0.138	0.387	0.183

**Visual Impairment**						
						
Eye sight trouble	0.792	0.792	0.488	0.467	0.470	0.448
Cataract	0.678	0.706	0.612	0.636	0.649	0.626
Glaucoma	0.668	0.673	0.523	0.515	0.566	0.567

**Cardiac symptoms/disease**						
						
Angina	0.619	0.602				
Ever had chest pain	0.674	0.674	0.835	0.829	0.838	0.866
Chest discomfort	0.411	0.387	0.466	0.476	0.344	0.393
Myocardial Infarction	0.885	0.797	0.68	0.702	0.737	0.733

**Respiratory symptoms/disease**						
						
Asthma	0.659	0.650	0.607	0.601	0.480	0.501
Bronchitis/emphysema	0.653	0.674	0.471	0.478	0.440	0.497
Short of breath on level walking	0.245	0.236	0.317	0.372	0.304	0.354
Increased cough/phlegm	0.582	0.546	0.491	0.533	0.550	0.546
Morning phlegm	0.621	0.596	0.509	0.538	0.540	0.525

**Psychological problems**						
						
Depression	0.583	0.524	0.156	0.228	0.365	0.335
Anxious or depressed/sad	0.773	0.8	2.174	1.501	0.721	0.792
Memory problems	0.208	0.207	0.107	0.174	0.367	0.346

**Physiological markers**						
						
Hypertensive (baseline>140/90)	0.754	0.258	1.853	0.084	1.282	1.063
Waist Hip Ratio (>/<0.85)	0.147	0.540	0.018	0.338	0.089	0.086
BMI (high)	0.149	0.464	0.045	0.722	0.039	0.068
Postural hypotension	0.339	0.111	0.120	-0.040	0.181	0.222
Sinus tachycardia	0.319	0.235	0.008	-0.060	0.058	0.016

**Other co-morbidities**						
						
Diabetes	0.353	0.382	0.305	0.267	0.253	0.188
Hypertension	0.567	0.467	0.542	0.647	0.507	0.591
Stroke	0.576	0.490	0.380	0.318	0.386	0.340
Stomach/peptic ulcers	-0.090	-0.077	-0.111	-0.073	-0.154	-0.092
Thyroid disease	-0.077	0.095	0.045	0.042	0.036	-0.059
Cancer	-0.144	-0.062	-0.011	0.009	-0.018	-0.005
Hearing trouble	-0.075	-0.208	-0.130	-0.095	-0.012	-0.044

**Figure 3:**
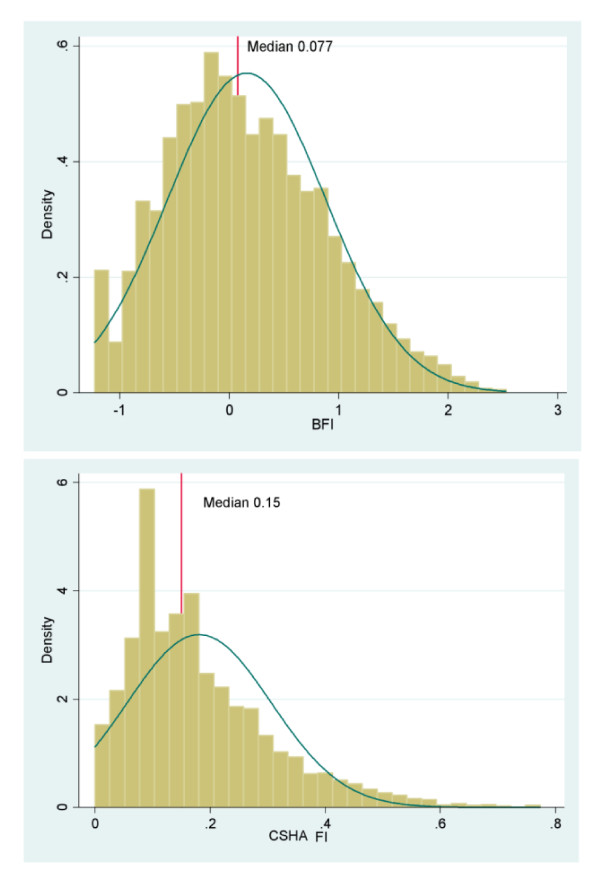
**A comparison of the distribution of the British FI and the CSHA FI in the BWHHS cohort of 4286 women**.

**Figure 4:**
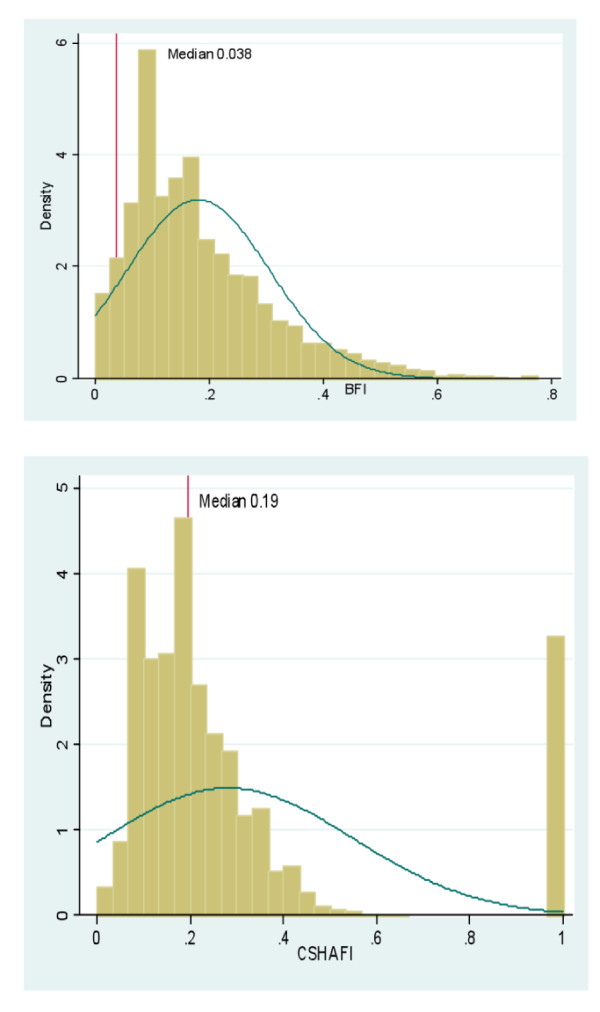
**A comparison of the distribution of the British FI and the CSHA FI in the MRC assessment study cohort of 11195 men and women**.

The distribution of frailty in BWHHS women and both men and women of the MRC assessment study, by age group and sex show that the BWHHS women (ages ranged from 60 to 79 years) in the older age group (over 75 years) had higher frailty scores i.e. were more frail compared to the younger age group (median scores 0.015 vs. 0.276). They also appeared to be more frail when compared to the MRC women, all of whom were over 75 years old (median scores 0.276 vs. 0.132). In the MRC women, the median frailty scores increased with age and when stratified, were higher in those in the older age groups of 80-84 years and 85 years and above, with scores of 0.213 and 0.578 respectively. The MRC men, whose scores also increased with age, were less frail compared to the women (median scores -0.811 vs. 0.132). A comparison of the distribution of the BFI and CSHA FI in both the BWHHS and MRC assessment study cohorts are shown in Figure 3 and Figure 4. The median score for the BFI was lower than the median score for the CSHA FI in both the BWHHS study cohort (0.07 vs. 0.15) (see Figure 3) and the MRC assessment study respondents (0.038 vs.0.19) (see Figure 4).

### Cox regression analysis

The British FI was a better predictor of all cause mortality in the women of the BWHHS cohort as shown in Table 4, when compared to the unweighted CSHA frailty index (age adjusted HR 1.7(95% C.I: 1.6,1.7) versus 1.4(95% C.I: 1.3,1.4).

**Table 4:** Hazard ratios for mortality per unit increase in frailty scores in 4286 BWHHS women

Frailty	Total(N)	British FI	CSHA FI
**Crude**	4286	1.8(1.7-2.0)	1.4(1.4,1.5)
**Age adjusted**	4286	1.7(1.6-1.8)	1.4(1.3,1.4)
Fully adjusted*	4280	1.4(1.3-1.5)	1.3(1.2,1.4)
p-value **		< 0.001	< 0.001

*fully adjusted for age, socioeconomic status (SES), smoking, alcohol intake, marital status, living alone and housing tenure.

**p value is for crude, age and fully adjusted hazard ratio (HR).

This was also true in both men and women of the MRC assessment study cohort (see Table 5), with frailty being a stronger predictor of mortality earlier on in the follow up period (between 0 to 2.5 years). The British FI was also a better predictor of the risk of hospital admission; fully adjusted HR 1.5(95% C.I: 1.4,1.6) vs. 1.3 (95% C.I: 1.2,1.3) as well as institutionalization; fully adjusted HR 1.6 (95% C.I: 1.4,1.8) vs. 1.3 (95% C.I: 1.2,1.4) in the MRC assessment study cohort (see Table 6). These predictions were independent of covariates such as age, sex, socioeconomic position scores, smoking, alcohol intake, living alone, marital status, housing tenure and social contact.

**Table 5:** Hazard ratios for mortality per unit increase in frailty scores in the MRC Assessment study

	Follow up time (years)								
	
	0-2.5			2.5-5.5			> 5.5		
Outcome	Hazard ratio (95% C.I)			Hazard ratio (95% C.I)			Hazard ratio (95% C.I)		
	Crude	Age	Full*	Crude	Age	Full*	Crude	Age	Full*
	**British FI**								
									
**All cause mortality**	2.0** (1.9,2.2)	1.9** (1.8,2.1)	1.8** (1.7,1.9)	1.7** (1.6,1.8)	1.6** (1.5,1.6)	1.5** (1.4,1.5)	1.5** (1.4,1.6)	1.4** (1.3,1.5)	1.4** (1.3,1.5)
									
	**CSHA FI (44 variables)**								
**All cause mortality**	1.6** (1.5,1.7)	1.5** (1.4,1.6)	1.5** (1.4,1.6)	1.4** (1.4,1.5)	1.3** (1.3,1.4)	1.3** (1.2,1.4)	1.3** (1.3,1.4)	1.2** (1.2,1.3)	1.3** (1.2,1.3)

*fully adjusted for age, sex, smoking, alcohol intake, marital status, living alone, social contact and housing tenure

**p value < 0.001

**Table 6:** Hazard ratios for hospitalization and institutionalization per unit increase in frailty scores in the MRC Assessment study

Outcome	Hazard ratio (95% C.I)		
	Crude	Age	Full*
	**British FI**		
**First hospital admission†**	1.6**(1.5-1.6)	1.5**(1.4,1.6)	1.5**(1.4,1.6)
**Institutionalization‡**	2.0**(1.8,2.2)	1.7**(1.5,1.9)	1.6**(1.4,1.8)

	**CSHA FI (44 variables)**		
**First hospital admission†**	1.4**(1.3,1.4)	1.3**(1.2,1.4)	1.3**(1.2,1.4)
**Institutionalization‡**	1.5**(1.4,1.6)	1.4**(1.2,1.5)	1.3**(1.2,1.4)

*fully adjusted for age, sex, smoking, alcohol intake, marital status, living alone, social contact and housing tenure.

**p value < 0.001

† refers to time to first hospital admission in the first two years of follow up.

‡ refers to time to institutionalization over a median time of 3.9 years of follow up

## Discussion

In order to better define the concept of frailty in older adults, we introduce a measurement model which was based on theoretical underpinnings of this concept, derived from an 'a priori' knowledge and research from existing literature [11,26,29,30] as well as statistical criteria. We used factor analysis (FA) to develop and test the hypothesis of frailty as a 'latent vulnerability' in older adults by incorporating all possible frailty indicators available to both datasets based on these criteria. Although the BFI is most related to the deficit accumulation index, its advantage over other measures is that it has weighted frailty indicators corrected for measurement error, which thus supports a more internally reliable measurement of frailty. EFA provided an initial latent structure of seven first order latent factors and CFA tested the hypothesis and confirmed the General specific model as the choice to form the conceptual basis for frailty in older adults. Using factor analysis, specific variance and random error is removed resulting in frailty, which is captured by the General factor (this factor represents the common variance between all the frailty indicators, thus capturing frailty). This model best reflects the association between frailty, its indicators and its underlying factors, in that particular indicators are explained by both a dominant general factor, (i.e. frailty), as well as seven specific factors, and these factors are mutually uncorrelated (see Figure 2). The implication is that frailty serves as the underlying factor that contributes to different forms of frailty indicators, and in addition, there are processes separate from this that contribute to the development of specific factors of visual impairment, respiratory disease/symptoms, cardiac disease/symptoms, physical ability, physiological markers, psychological problems and co-morbid disease, which vary independently of frailty. By contrast, in the 2^nd ^order model, frailty was seen to drive/subsume all the factors/dimensions acting as a single broad, coherent construct broken down into increasingly specific factors and indicators (see Additional file 1: Supplementary figure F2: Second order model).

In the 1^st ^order model, frailty was represented by each of the seven specific factors that were correlated to each other (see Additional file 1: Supplementary figure F1: First order model).

On a conceptual level, these models (1^st ^and 2^nd ^order) do not fit in with the idea of frailty. Not all the specific factors need to be present for an individual to be considered frail, as implied by the second order model. For example, an elderly diabetic with 'eyesight trouble' and 'difficulty in going out' may still be considered frail despite not having other co-morbidities, cardio-respiratory disease or symptoms. The problem with the 1^st ^order model was that the factors do not necessarily need to be correlated to one another for frailty to occur (see Additional file 1*to compare the models*).

External/exogenous to this measurement model were socioeconomic status (SES) indicators such as income, education, social class, marital status, lifestyle indicators as well as social contact. As frailty is likely to be socially patterned [26], SES was expected to have a causally influence on frailty[39]. Hence frailty can be thought of as a mixed (reflective and formative) construct, that is reflected in the binary frailty indicators, but also driven by SES status[40] among other external/exogenous forces.

Although some measures of frailty were developed by defining and quantifying the construct through data driven approaches, they were not developed appropriately for the binary/ordinal nature of the data. Other population studies have developed frailty measures using principal component analysis (PCA) [13,41,42]. Unlike one particular study that looked for sub dimensions of a pre-existing physical phenotype of frailty[42], our measure used all known and easily available frailty indicators in the datasets so as to fulfil its multi-dimensional concept. FA is used to identify the structure underlying all the frailty indicators and provides more internal reliability to the measure by controlling for measurement error, as it analyzes only the variability in an indicator that is shared among the other indicators (common variance without error or unique variance) while PCA assumes that all variability in an indicator should be used in the analysis.

In both datasets, a majority of indicators represented by physical ability were ones that best explained frailty. This supports the theory that frailty is identified through characteristics directly related to physical function [26]. The analysis also highlighted the importance of 'shortness of breath on level walking' as a more important frailty indicator than diagnosed respiratory diseases. Similarly, reports of symptoms such as 'ever having chest pain/chest discomfort' had higher factor loadings than having had a myocardial infarction. These higher loadings of self reported symptoms compared to diagnosed conditions might reflect that the diagnosed diseases were already under control or treated in our respondents. Although co-morbidities featured strongly in some existing measures [13,43], our model focused specifically on diseases such as myocardial infarction, angina, stroke, diabetes, peptic ulcers and hypertension.

Whilst frailty has been conceptualized as a wasting syndrome with weight loss as a key component, it was also explained by having a high BMI and a high waist to hip ratio in both cohorts. This finding supports a recent study that showed increased levels of frailty among those with low and very high BMI and within each BMI category; those with a high waist circumference were significantly more frail[44]. In view of the rise in obesity in older populations, lifestyle modifications incorporating a healthy diet and regular exercise should be an important agenda in the prevention of frailty and its adverse outcomes. However these efforts should not merely target the usual overweight/obese older adults but those who exhibit signs of central obesity, regardless of BMI category.

**Figure 5:**
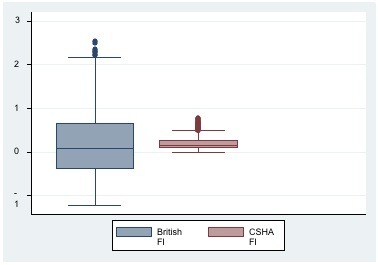
**Graph-box showing median and inter-quartile ranges of the British FI and CSHA FI in 4286 BWHHS women**.

**Figure 6:**
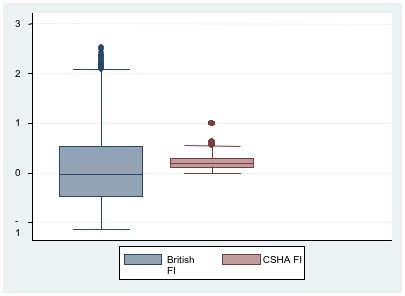
**Graph-box showing median and inter-quartile ranges of the British FI and CSHA FI in 11195 MRC assessment study respondents**.

Comparisons between the British frailty index (FI) and the well validated CSHA frailty index showed that the British FI had greater variance in the distribution of scores compared to the CSHA FI (see Figure 5 and 6). Hence, the British FI would serve as a better population metric than the CSHA FI as it enables those people with varying degrees of frailty from low to mild, moderate and severe to be better distinguished over a wider range of scores. The British FI was a better predictor of all cause mortality than CSHA FI in both cohorts independent of similar potential confounders. It was also a better estimate of the respondents' increased risk of hospital admission per unit of frailty score than both versions of the CSHA index. However, the outcome of hospitalization in this study only involved the time to first hospital admission for each respondent during the whole follow up period of the MRC assessment study. These results suggest that further analyses into those with multiple admissions would indeed be of value in classifying the frailest among this population as it is a common problem among older people and drive a large part of the burden and costs associated with frailty. Institutionalized older people are often labeled as frail and hence, the risk of institutionalization has become a recognized frailty adverse outcome. Using the British FI, frailty also estimated a better increased and independent risk of institutionalization, per unit score than the CSHA index. These findings explain the advantage of the British frailty measure over the CSHA index; in that it is a reduced measure that corrects for measurement error and assigns relative weights in the association of each indicator with frailty. In developing this measure, the weighted latent variables that best explained frailty were captured, excluding those that did not. This resulted in a measure that attempts to measure frailty itself as opposed to being an indicator of an older person's global health status. As the two different measures of frailty are based on different theoretical constructs, they would certainly capture different groups of older people. Hence the results above suggest that the British FI would serve as a better predictor of adverse outcomes in community dwelling older people than an unweighted and additive type of index.

The strength of this study lie in the construction of a measurement model of frailty in a large representative cohort of British women and its replication in a further large cohort representative of the British community-dwelling older population of men and women, using variables that were direct inputs from the respondents, including both objective and subjective attributes. FA enabled the identification of latent dimensions of frailty that may not have been apparent from direct observation of the data. This also enabled us to develop a reliable measure that translated into a frailty score for use in future analyses. Although the identification of these seven factors were in keeping with other measures based on similar domains[8,12,21], the development of a tool (using indicators which are both weighted and corrected for measurement error) lends added credibility to it being a more reliable measurement of frailty. The reliability or internal consistency of the 'General Specific' model was shown by the goodness of fit of the confirmatory factor analysis. The validation of the model as a measurement of frailty was reaffirmed when the same model was tested in a larger independent cohort of the MRC assessment study whose respondents were older of both sexes. The higher weighted frailty indicators provide more precise information than is currently recognized, as to which cluster of frailty indicators are important in identifying frailty in older people. Furthermore, it provides important information about the survival prediction of older people over long follow up periods which makes it a good prognostic tool that would aid in the planning and allocation of health care services for them.

A limitation of this study is that as the majority of the participants are older Caucasians, our results may not necessarily be generalisable to younger adults or other ethnic groups. The BWHHS study respondents were those who were able to attend the interview and medical examination at baseline suggests that they were relatively less frail compared to non-responders. Therefore, this study cohort may underestimate the degree of frailty among the population it derived its sample from. Another limitation is that the frailty indicators used were derived from self-reports of symptoms/disease at baseline; hence it is not a dynamic measure of frailty. We concentrated on only complete cases but found similar findings for those with missing data. Although indicators used were based on known indicators from existing measures, we were limited to those available in both datasets.

In this paper we wish to highlight the additional contribution of the BFI to the existing concepts and measures of frailty from a purely measurement point of view. In its current form, the BFI is still in the early stage of development and will need further refinement. Although it is ready for use in a research setting, its clinical application (as with any other scale) will require further appropriate models in order to establish reliable cut off points. The refined version would be able to include missing data with fewer, higher weighted indicators which are controlled for measurement error. These indicators represent each of the seven latent factors associated with frailty, which would be translated into a short answer questionnaire, making it more amenable for use in a clinical setting. Existing measures suggests two perspectives on frailty; its use as an indicator of health and its use as a clinical tool. In constructing the BFI, we recommend that the measurement of frailty should include both perspectives.

## Conclusion

This study provides a better understanding of the widely held view of the multi-dimensional domains of frailty and its concept as a latent vulnerability in older people. It does so by providing a more reliable method of its measurement that demonstrates better validity particularly in relation to serious adverse outcomes when compared to a widely known frailty index. This new frailty measure may provide further opportunities and modifiable strategies for prevention and health promotion at a population level as well improved detection, treatment and intervention of frailty in older people at a clinical level. Future work will involve translating this model into a simple index that is easy and non invasive for use in a primary care setting.

## Competing interests

The authors declare that they have no competing interests.

## Authors' contributions

All authors developed the study's aim, design and managed its data. SK performed the statistical analysis and drafted the manuscript. GP advised and participated in the statistical analysis. All authors have read and approved the final manuscript.

## Supplementary Material

Additional File 1 **Supplementary tables and figures**. **SUPPLEMENTARY TABLE S1: ALL FRAILTY INDICATORS (Non-Invasive)**. All the non- invasive frailty indicators included in the factor analysis that was derived from existing literature and available to both cohorts. **SUPPLEMENTARY TABLE S2: ADDITIONAL FRAILTY INDICATORS (Invasive)**. Additional invasive frailty indicators not included in the factor analysis. **Variables used to derive the CSHA FI using the BWHHS study cohort**. This is a list of 51 variables from the BWHHS study used to derive the CSHA FI. **Variables used to derive the CSHA FI using the MRC assessment study cohort**. This is a list of 44 variables from the MRC assessment study cohort used to derive the CSHA FI. **Supplementary figure F1: First order model**. Figure illustrating frailty as a first order model derived from factor analysis. **Supplementary figure F2: Second order model**. Figure illustrating frailty as a second order model derived from factor analysis. Click here for file

## References

[B1] Woods NF LaCroix AZ Gray SL Aragaki A Cochrane BB Brunner RL Masaki K Murray A Newman AB Frailty: emergence and consequences in women aged 65 and older in the Women's Health Initiative Observational Study. [see comment] Journal of the American Geriatrics Society 2005 53 1321 1330 10.1111/j.1532-5415.2005.53405.x 16078957

[B2] Fried LP Tangen CM Walston J Newman AB Hirsch C Gottdiener J Seeman T Tracy R Kop WJ Burke G McBurnie MA Frailty in older adults: evidence for a phenotype Journal of Gerontology: Medical Sciences 2001 56 M146 156 10.1093/gerona/56.3.m146 11253156

[B3] Ensrud KE Ewing SK Taylor BC Fink HA Stone KL Cauley JA Tracy JK Hochberg MC Rodondi N Cawthon PM Frailty and risk of falls, fracture, and mortality in older women: The study of Osteoporotic fractures Journal of Gerontology: Medical Sciences 2007 62 744 751 10.1093/gerona/62.7.744 17634322

[B4] Nourhashemi F Andrieu S Gillette-Guyonnet S Vellas B Albarede JL Grandjean H Instrumental activities of daily living as a potential marker of frailty: a study of 7364 community-dwelling elderly women (the EPIDOS study) Journal of Gerontology: Medical Sciences 2001 56 M448 453 10.1093/gerona/56.7.m448 11445604

[B5] Rockwood K Howlett S MacKnight C Beattie B Bergman H Hebert R Hogan D Wolfson C McDowell I Prevalence, attributes, and outcomes of fitness and frailty in community-dwelling older adults: Report from the Canadian Study of Health and Aging Journal of Gerontology: Medical Sciences 2004 59 1310 1317 10.1093/gerona/59.12.1310 15699531

[B6] Rockwood K Song XW MacKnight C Bergman H Hogan DB McDowell I Mitnitski A A global clinical measure of fitness and frailty in elderly people Canadian Medical Association Journal 2005 173 489 495 10.1503/cmaj.050051 PMC1188185 16129869

[B7] Cawthon PM Marshall LM Michael Y Dam T-T Ensrud KE Barrett-Connor E Orwoll ES Osteoporotic Fractures in Men Research G Frailty in older men: prevalence, progression, and relationship with mortality Journal of the American Geriatrics Society 2007 55 1216 1223 10.1111/j.1532-5415.2007.01259.x 17661960

[B8] Saliba D Elliott M Rubenstein LZ Solomon DH Young RT Kamberg CJ Roth C MacLean CH Shekelle PG Sloss EM Wenger NS The vulnerable elders survey: A tool for identifying vulnerable older people in the community Journal of the American Geriatrics Society 2001 49 1691 1699 10.1046/j.1532-5415.2001.49281.x 11844005

[B9] Puts MTE Lips P Deeg DJH Static and dynamic measures of frailty predicted decline in performance-based and self-reported physical functioning Journal of Clinical Epidemiology 2005 58 1188 1198 10.1016/j.jclinepi.2005.03.008 16223663

[B10] Brown I Renwick R Raphael D Frailty: constructing a common meaning, definition, and conceptual framework International Journal of Rehabilitation Research 1995 18 93 102 7665266

[B11] Rockwood K Hogan DB Macknight C Conceptualisation and Measurement of Frailty in Elderly People Drugs and Aging 2000 17 295 302 10.2165/00002512-200017040-00005 11087007

[B12] Strawbridge WJ Shema SJ Balfour JL Higby HR Kaplan GA Antecedents of frailty over three decades in an older cohort J Gerontol B Psychol Sci Soc Sci 1998 53 S9 16 10.1093/geronb/53b.1.s9 9469175

[B13] Schuurmans H Steverink N Lindenberg S Frieswijk N Slaets JPJ Old or frail: what tells us more? Journal of Gerontology: Medical Sciences 2004 59 M562 565 10.1093/gerona/59.9.m962 15472162

[B14] Studenski S Hayes RP Leibowitz RQ Bode R Lavery L Walston J Duncan P Perera S Clinical Global Impression of Change in Physical Frailty: development of a measure based on clinical judgment Journal of the American Geriatric Society 2004 52 1560 1566 10.1111/j.1532-5415.2004.52423.x 15341562

[B15] Syddall H Cooper C Martin F Briggs R Aihie A Sayer Is grip strength a useful single marker of frailty? Age & Ageing 2003 32 650 656 10.1093/ageing/afg111 14600007

[B16] Boxer RS Wang Z Walsh SJ Hager D Kenny AM The utility of the 6-minute walk test as a measure of frailty in older adults with heart failure American Journal of Geriatric Cardiology 2008 17 7 12 10.1111/j.1076-7460.2007.06457.x 18174754

[B17] Weiner DK Duncan PW Chandler J Studenski SA Functional reach: a marker of physical frailty Journal of the American Geriatric Society 1992 40 203 207 10.1111/j.1532-5415.1992.tb02068.x 1538035

[B18] Leng S Chaves P Koenig K Walston J Serum interleukin-6 and hemoglobin as physiological correlates in the geriatric syndrome of frailty: a pilot study Journal of the American Geriatric Society 2002 50 1268 1271 10.1046/j.1532-5415.2002.50315.x 12133023

[B19] Bartali B Frongillo EA Bandinelli S Lauretani F Semba RD Fried LP Ferrucci L Low Nutrient Intake Is an Essential Component of Frailty in Older Persons Journal of Gerontology: Medical Sciences 2006 61 589 593 10.1093/gerona/61.6.589 PMC2645617 16799141

[B20] Mitnitski AB Song X Rockwood K The estimation of relative fitness and frailty in community-dwelling older adults using self-report data Journal of Gerontology: Medical Sciences 2004 59 M627 M632 10.1093/gerona/59.6.m627 15215283

[B21] Rockwood K Minitski A Song X Steen N Skoog I Long-term Risks of Death and Institutionalization of Elderly People in Relation to Deficit Accumulation at Age 70 J AM Geriatr Soc 2006 54 975 979 10.1111/j.1532-5415.2006.00738.x 16776795

[B22] Klein BEK Klein R Knudtson MD Lee KE Frailty, morbidity and survival Archives of Gerontology and Geriatrics 2005 41 141 149 10.1016/j.archger.2005.01.002 16085065

[B23] Goggins WB Woo J Sham A Ho SC Frailty index as a measure of biological age in a Chinese population Journal of Gerontology: Medical Sciences 2005 60 1046 1051 10.1093/gerona/60.8.1046 16127111

[B24] Darlington RB Factor Analysis http://www.psych.cornell.edu/Darlington/factor.htm accessed August 2008

[B25] Tabachnick BG Fidell LS Using multivariate statistics 2007 Fifth

[B26] Walston J Hadley EC Ferrucci L Guralnik JM Newman AB Studenski SA Ershler WB Harris T Fried LP Research agenda for frailty in older adults: toward a better understanding of physiology and etiology: summary from the American Geriatrics Society/National Institute on Aging Research Conference on Frailty in Older Adults Journal of the American Geriatrics Society 2006 54 991 1001 10.1111/j.1532-5415.2006.00745.x 16776798

[B27] Rockwood K Minitski A Frailty in Relation to the Accumulation of Deficits Journal of Gerontology: MEDICAL SCIENCES 2007 62A 722 727 10.1093/gerona/62.7.722 17634318

[B28] Lawlor DA Patel R Ebrahim S Association between falls in elderly women and chronic diseases and drug use: a cross sectional study British Medical Journal 2003 327 1 6 10.1136/bmj.327.7417.712 PMC200802 14512478

[B29] Strawbridge WJ Shema SJ Balfour JL Higby HR Kaplan GA Antecedents of frailty over three decades in an older cohort Journal of Gerontology: Social Sciences 1998 53 S9 S16 10.1093/geronb/53b.1.s9 9469175

[B30] Fried LP Ferrucci L Darer J Williamson JD Anderson G Untangling the Concepts of Disability, Frailty, and Comorbidity: Implications for Improved Targeting and Care Journal of Gerontology: Medical Sciences 2004 59 255 263 10.1093/gerona/59.3.m255 15031310

[B31] Fletcher AE Jones DA Bulpitt CJ Tulloch AJ The MRC trial of assessment and management of older people in the community: objectives, design and interventions BMC Health Services Research 2002 2 10.1186/1472-6963-2-21 PMC134467 12398790

[B32] Jarman B Underprivileged areas: validation and distribution of scores British Medical Journal (Clinical Research Edition) 1984 289 1587 1592 10.1136/bmj.289.6458.1587 PMC1443922 6439333

[B33] Kline RB Principles and practice of structural equation modeling 1998 New York: Guilford Press

[B34] Field A Discovering Statistics using SPSS for Windows 2000 London-Thousand Oaks-New Delhi: Sage publications

[B35] Hu L Bentler PM Cutoff criteria for fit indexes for covariance structure analysis:conventional criteria versus new alternatives Structural Equation Modeling 1999 6 1 55

[B36] Gu D Dupre ME Sautter J Zhu H Liu Y Yi Z Frailty and mortality among Chinese at advanced ages Journal of Gerontology: Social Sciences 2009 64 279 289 10.1093/geronb/gbn009 PMC2655172 19196691

[B37] Woo J Goggins W Sham A Ho SC Social determinants of frailty Gerontology 2005 51 402 408 10.1159/000088705 16299422

[B38] Kulminski A Ukraintseva SV Akushevich I Arbeev KG Land K Yashin AI Accelerated accumulation of health deficits as a characteristic of aging Experimental Gerontology 2007 42 963 970 10.1016/j.exger.2007.05.009 PMC2096614 17601693

[B39] Andrew MK Minitski AB Rockwood K Social vulnerability, frailty and mortality in elderly people PL oS ONE 2008 3 e2232 10.1371/journal.pone.0002232 PMC2375054 18493324

[B40] Lubke GH Muthen B Investigating Population Heterogeneity with Factor Mixture Models Psychological Methods 2005 10 21 39 10.1037/1082-989X.10.1.21 15810867

[B41] Speechley M Tinetti M Falls and Injuries in Frail and Vigorous Community Elderly Persons Journal of the American Geriatrics Society 1991 39 46 52 10.1111/j.1532-5415.1991.tb05905.x 1987256

[B42] Sarkisian CA Gruenewald TL John W Boscardin Seeman TE Preliminary evidence for subdimensions of geriatric frailty: the MacArthur study of successful aging Journal of the American Geriatric Society 2008 56 2292 2297 10.1111/j.1532-5415.2008.02041.x PMC2754409 19016933

[B43] Rockwood K Mitnitski A Song XW Steen B Skoog I Long-term risks of death and institutionalization of elderly people in relation to deficit accumulation at age 70 Journal of the American Geriatrics Society 2006 54 975 979 10.1111/j.1532-5415.2006.00738.x 16776795

[B44] Hubbard RE Lang IA Llewellyn DJ Rockwood K Frailty, Body Mass Index, and Abdominal Obesity in Older People J Gerontol A Biol Sci Med Sci 2010 65 4 377 81 10.1093/gerona/glp186 19942592

