# Supplementary material for: A reliable measure of frailty for a community dwelling older population
Source: Health Qual Life Outcomes. 2010 Oct 28;8:123. doi: 10.1186/1477-7525-8-123 (PMC2988728; doi:10.1186/1477-7525-8-123)
Supplement: Additional File 1 — Supplementary tables and figures. SUPPLEMENTARY TABLE S1: ALL FRAILTY INDICATORS (Non-Invasive). All the non- invasive frailty indicators included in the factor analysis that was derived from existing literature and available to both cohorts. SUPPLEMENTARY TABLE S2: ADDITIONAL FRAILTY INDICATORS (Invasive). Additional invasive frailty indicators not included in the factor analysis. Variables used to derive the CSHA FI using the BWHHS study cohort. This is a list of 51 variables from the BWHHS study used to derive the CSHA FI. Variables used to derive the CSHA FI using the MRC assessment study cohort. This is a list of 44 variables from the MRC assessment study cohort used to derive the CSHA FI. Supplementary figure F1: First order model. Figure illustrating frailty as a first order model derived from factor analysis. Supplementary figure F2: Second order model. Figure illustrating frailty as a second order model derived from factor analysis. [file 1477-7525-8-123-S1.DOC]

**Additional file 1:**

**SUPPLEMENTARY TABLE S1: ALL FRAILTY INDICATORS (Non-Invasive)**

| **ALL FRAILTY INDICATORS USED FOR**  **IN FACTOR ANALYSIS*** | **BFI**  **(weighted)** | **CSHA**  **Index (unweighted)** |
| --- | --- | --- |
| Living with someone else? | X | X |
| Any contact with others i.e. relatives, friends, siblings, children, neighbours? | X | X |
| How would you describe your health at present? | X | X |
| Have you had a fall in past year? | **√** | **√** |
| Compared with your activity level 3 years ago, are you doing more, same or less? | **√** | **√** |
| Do you have problems washing or dressing? (no problem, some problem, unable to wash and dress) | **√** | **√** |
| Is your present state of health causing you problems with household chores? | **√** | **√** |
| Difficulty in carrying out activity on their own: going up and downstairs | **√** | **√** |
| Difficulty in carrying out activity on their own: Walking about | **√** | **√** |
| Difficulty in carrying out activity on their own: Going out of the house | **√** | **√** |
| Difficulty in carrying out activity on their own: Walking 400 yards | X | X |
| Do you have trouble with your hearing? | X | **√** |
| Do you have trouble with your eyesight? (not simply needing specs) | **√** | **√** |
| Compared to five years ago, is your memory: improved, same, almost as good, worse, much worse? Dementia on medical exam. | **√** | **√** |
| Your health over all: are you anxious or depressed, not depressed – moderately, extremely. | **√** | **√** |
| Do you smoke cigarettes currently? If so, how many? | X | X |
| Would you describe your intake as: (1.daily, most days, 2.weekends only, 3.once/twice a month, 4. special occasions) | X | X |
| Type of accommodation? (owner occupier, renting from local authority, renting privately, other) | X | X |
| Do your ankles swell up regularly? | X | **√** |
| Do you ever have any pain or discomfort in your chest? | **√** | **√** |
| Have you ever had a severe pain across the front of your chest lasting for half an hour or more? | **√** | **√** |
| Do you usually bring up phlegm (spit) from your chest first thing in the morning in the winter? | **√** | **√** |
| Do you bring up phlegm on most days as much as 3 months in the winter each year? | X | X |
| In the past four years, have you ever had a period of increased cough and phlegm lasting for 3 weeks or more? | **√** | **√** |
| Does your chest often sound wheezy (on most days or nights?) | X | X |
| Do you get short of breath with other people of your own age on level ground? | **√** | **√** |
| How would you describe your health at present? | X | X |
| Have you ever been told by a doctor that you have or have had asthma? | **√** | **√** |
| Have you ever been told by a doctor that you have or have had bronchitis or emphysema? | **√** | **√** |
| Have you ever been told by a doctor that you have or have had arthritis? | **√** | **√** |
| Have you ever been told by a doctor that you have or have had high blood pressure? | **√** | **√** |
| Have you ever been told by a doctor that you have or have had thyroid disease? | **√** | **√** |
| Have you ever been told by a doctor that you have or have had a cataract? | **√** | **√** |
| Have you ever been told by a doctor that you have or have had glaucoma? | **√** | **√** |

Continued…

Continued…

**SUPPLEMENTARY TABLE S1: ALL FRAILTY INDICATORS (Non-Invasive)**

| **ALL FRAILTY INDICATORS USED FOR IN FACTOR ANALYSIS*** | **BFI**  **(weighted)** | **CSHA**  **Index (unweighted)** |
| --- | --- | --- |
| Have you ever been told by a doctor that you have or have had gout? | X | **√** |
| Have you ever been told by a doctor that you have or have had depression? | **√** | **√** |
| Have you ever been told by a doctor that you have or have had diabetes? | **√** | **√** |
| Have you ever been told by a doctor that you have or have had gastric or peptic ulcer? | **√** | **√** |
| Have you ever been told by a doctor that you have or have had heart attack (MI)? | **√** | **√** |
| Have you ever been told by a doctor that you have or have had angina? | **√** | **√** |
| Have you ever been told by a doctor that you have or have had a stroke? | **√** | **√** |
| Have you ever been told by a doctor that you have or have had cancer? | **√** | **√** |
| Have you ever fractured your hip? | X | **√** |
| Cardiovascular disease (diagnosed angina, MI, stroke) | **√** | **√** |
| Body mass index: high or low | **√** | **√** |
| Postural hypotension: According to consensus definition | **√** | **√** |
| Hypertensive (>140/90) | **√** | **√** |
| Waist hip ratio (>/<0.85 | **√** | **√** |
| Sinus tachycardia (>100 bpm) | **√** | **√** |

*All indicators listed were ones originally included in the factor analysis from which 35 indicators were derived and confirmed by the data.

**SUPPLEMENTARY TABLE S2: ADDITIONAL FRAILTY INDICATORS (Invasive)**

| **BLOOD MARKERS AS FRAILTY INDICATORS** | **BFI**  **(weighted)** | **CSHA**  **Index (unweighted)** |
| --- | --- | --- |
| Low Haemoglobin | X | **√** |
| High Cholesterol | X | **√** |
| Low Albumin | X | **√** |
| High Creatinine | X | **√** |
| High Glucose | X | **√** |

**Variables used to derive the CSHA FI using the BWHHS study cohort**

**CSHA FI (51 variables)**

1. Low Haemoglobin
2. High cholesterol
3. Low albumin
4. High creatinine
5. High glucose
6. Low BMI
7. High BMI
8. Waist hip ratio
9. High blood pressure (measured)
10. Orthostatic hypotension (measured)
11. Sinus Tachycardia (>100 bpm)
12. Eye sight trouble
13. Hearing trouble
14. Cataract
15. Glaucoma
16. Asthma
17. Arthritis
18. Angina
19. Ankle oedema
20. Bronchitis
21. Cancer
22. Cerebrovascular disease
23. Anxious or depressed
24. Depression
25. Diabetes Mellitus
26. Gout
27. High blood pressure (self report of diagnosed)
28. Falls
29. Hip fracture
30. Memory problems/dementia
31. Myocardial infarction
32. Stroke
33. Thyroid disease
34. Ulcers
35. Unable to walk out of house/difficulty in going out
36. Difficulty in walking about
37. Difficulty walking 400 yards
38. Difficulty going up and down stairs
39. Difficulty doing household chores
40. Difficulty washing and dressing oneself
41. Status activity level
42. Shortness of breath
43. Increased cough
44. Increased and often wheeze
45. Morning phlegm
46. Most days phlegm
47. Ever had chest pain
48. Chest discomfort
49. Chest pain
50. On level pain
51. On uphill pain

**Variables used to derive the CSHA FI using the MRC assessment study cohort**

**CSHA FI (44 variables)**

1. Low Haemoglobin
2. High cholesterol
3. Low albumin
4. High creatinine
5. High glucose
6. Low BMI
7. High BMI
8. Waist hip ratio
9. High blood pressure (measured)
10. Orthostatic hypotension (measured)
11. Sinus Tachycardia (>100 bpm)
12. Eye sight trouble
13. Hearing trouble
14. Cataract
15. Glaucoma
16. Asthma
17. Arthritis
18. Emphysema
19. Cancer
20. Anxious or depressed
21. Depression
22. Diabetes Mellitus
23. Hip fracture
24. High blood pressure (self report of diagnosed)
25. Falls
26. Memory problems/dementia
27. Myocardial infarction
28. Stroke
29. Thyroid disease
30. Ulcers
31. Unable to walk out of house/difficulty in going out
32. Difficulty going up and down stairs
33. Difficulty doing household chores
34. Difficulty washing and dressing oneself
35. Status activity level
36. Shortness of breath
37. Increased cough
38. Increased and often wheeze
39. Morning phlegm
40. Most days phlegm
41. Ever had chest pain
42. Chest discomfort
43. On level pain
44. On uphill pain

**Supplementary Figure F1: First order model**


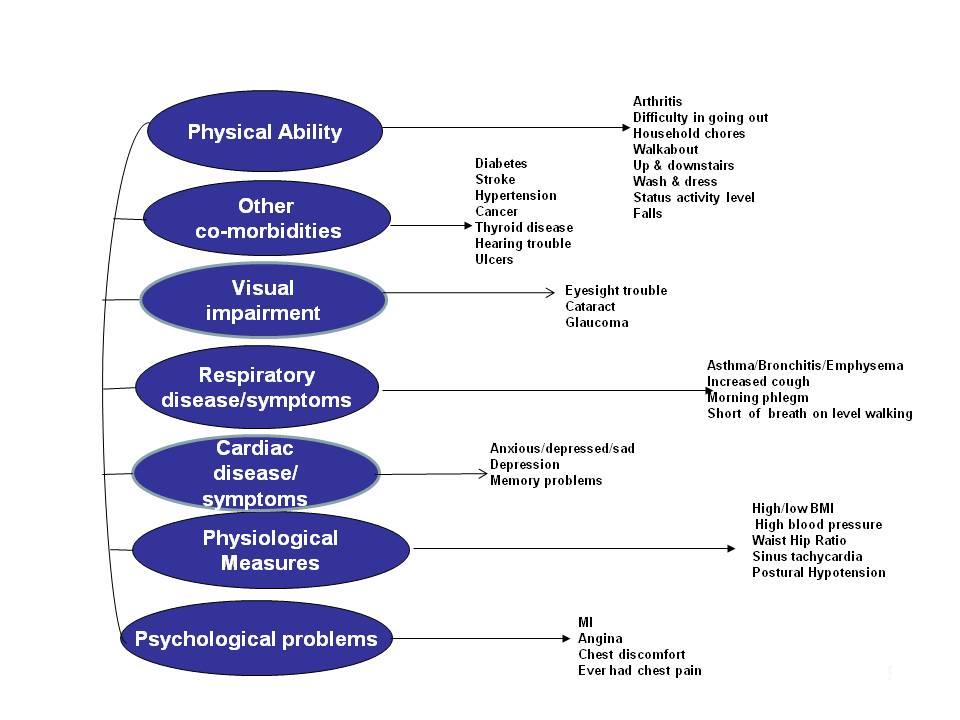


**Supplementary Figure F2: Second order model**
